# BPI-ANCA Provides Additional Clinical Information to Anti-*Pseudomonas* Serology: Results from a Cohort of 117 Swedish Cystic Fibrosis Patients

**DOI:** 10.1155/2015/947934

**Published:** 2015-07-26

**Authors:** Ulrika Lindberg, Malin Carlsson, Thomas Hellmark, Mårten Segelmark

**Affiliations:** ^1^Department of Clinical Sciences in Lund, Section of Respiratory Medicine and Allergology, Lund University and Skane University Hospital, 221 85 Lund, Sweden; ^2^Department of Clinical Sciences in Lund, Section of Nephrology, Lund University, 221 85 Lund, Sweden; ^3^Department of Medicine and Health, Linköping University, 581 83 Linköping, Sweden; ^4^Department of Nephrology UHL, County Council of Östergötland, 581 83 Linköping, Sweden

## Abstract

Patients with cystic fibrosis (CF) colonized with* Pseudomonas aeruginosa* (*P. aeruginosa*) have worse prognosis compared with patients who are not. BPI-ANCA is an anti-neutrophil cytoplasmic antibody against BPI (bactericidal/permeability increasing protein) correlating with* P. aeruginosa *colonization and adverse long time prognosis. Whether it provides additional information as compared to standard anti-*P. aeruginosa* serology tests is not known. 117 nontransplanted CF patients at the CF centre in Lund, Sweden, were followed prospectively for ten years. Bacterial colonisation was classified according to the Leeds criteria. IgA BPI-ANCA was compared with assays for antibodies against alkaline protease (AP), Elastase (ELA), and Exotoxin A (ExoA). Lung function and patient outcome, alive, lung transplanted, or dead, were registered. BPI-ANCA showed the highest correlation with lung function impairment with an *r*-value of 0.44. Forty-eight of the 117 patients were chronically colonized with* P. aeruginosa*. Twenty of these patients experienced an adverse outcome. Receiver operator curve (ROC) analysis revealed that this could be predicted by BPI-ANCA (AUC = 0.77), (*p* = 0.002) to a better degree compared with serology tests. BPI-ANCA correlates better with lung function impairment and long time prognosis than anti-*P. aeruginosa* serology and has similar ability to identify patients with chronic* P. aeruginosa*.

## 1. Introduction

*Pseudomonas aeruginosa* (*P. aeruginosa*) is the most significant pathogen in cystic fibrosis (CF) [1]. Chronic infection with* P. aeruginosa* develops in most CF patients and in many CF clinics 60–80% of the adult patients are chronically infected with* P. aeruginosa* [2]. In a majority of patients, chronic infection is preceded by intermittent colonization.

The analysis of antibodies against* P. aeruginosa* started in the 1970s, with Hoibys work on* P. aeruginosa* precipitins [3]. Different commercial tests are now available. Measuring antibodies against* P. aeruginosa* has been shown to be useful in characterizing patients with different infection status and elevated titers have been shown to be a risk factor for developing chronic* P. aeruginosa* infection [4, 5]. Serology may also be useful to monitor response to therapy [6]. Early intervention against* P. aeruginosa* can prevent some of the patients from becoming chronically infected [7] and thus it is essential to detect the bacteria in the airways as early as possible. This can be a diagnostic problem in nonsputum producing patients, mainly children, as the clinician usually has to rely on cultures from oropharyngeal swabs. Serum antibodies may be detected before the organism is isolated from respiratory samples [8] although there is still some controversy about this [9]. A rise in antibody titres indicates probable infection and eradication treatment may be initiated even in the absence of microbiological detection of* P. aeruginosa* [10] although antibodies are not recommended as the only way of diagnosing a new* P. aeruginosa *infection [6].

Recently a review article about serum antibodies to* P. aeruginosa* in CF was published [11] and the authors found that studies show a good correlation between anti-*Pseudomonas* antibody titers and clinical status and that* P. aeruginosa* serology can be useful to evaluate the colonization/infection status of the patient. The review authors conclude that there is support to suggest the incorporation of* P. aeruginosa* serology in the follow-up routine of CF patients.

Bactericidal/permeability increasing (BPI) protein is found in the azurophilic granules of neutrophil granulocytes. BPI has a potent antimicrobial activity against Gram-negative bacteria, such as* P. aeruginosa*, by neutralising the endotoxin and by playing a part in opsonization of the bacteria [12–18]. Anti-neutrophil cytoplasmic antibodies (ANCA) with BPI specificity have been identified in different diseases associated with Gram-negative bacteria, such as inflammatory bowel diseases (IBD) and primary sclerosing cholangitis [19], and are frequently present in CF patients [16, 20, 21]. Earlier publications from our research group have shown a correlation between the presence of both IgA and IgG BPI-ANCA and reduced lung function in CF [22], with slightly better correlation for IgA BPI-ANCA. A high level of BPI-ANCA was associated with more severe lung disease when measured with both radiology and spirometry [21, 23]. The mechanism behind BPI-ANCA production is a poorly understood process.

The relation between BPI-ANCA and different* P. aeruginosa *serologies has not been studied so far and it is not known if BPI-ANCA yields the same set of information as* P. aeruginosa *serology tests. In a previous study, we found patients with chronic* P. aeruginosa* colonization who remained ANCA-negative for over a decade suggesting that BPI-ANCA shows something different than* Pseudomonas *colonization/infection or* P. aeruginosa *serology tests [24]. This study was performed to further analyse how BPI-ANCA differs from* P. aeruginosa* serology as our earlier investigations indicate that BPI-ANCA has a potential clinical use as a prognostic factor in CF. The objective of this study was to compare BPI-ANCA with* P. aeruginosa* serology with respect to lung function impairment, prediction of outcome, detection of chronic* P. aeruginosa* colonization, and prediction of future colonization.

## 2. Patients and Methods

### 2.1. Patients

Out of the 135 patients registered at the CF centre at Skane University Hospital in Lund in 2001 all nontransplanted patients (*n* = 127) were eligible for the study and 121 of these patients were included during the inclusion period (October 2001 through March 2003). Four patients were later excluded because of missing serological data (*n* = 3) or missing microbiological data (*n* = 1). No patient was lost to follow-up. The Ethical Committee at Lund University approved the study and all participants gave their written informed consent before inclusion. The CF diagnosis was confirmed genetically as part of the clinical routine and the results of mutation analyses as well as all other clinical data were obtained from patient records. Initial data, including IgA BPI-ANCA, anti-*Pseudomonas* serology, and lung function, was registered at study start. A follow-up, measuring lung function and registering clinical outcome (alive, lung transplanted, or deceased), was performed ten years after inclusion.

### 2.2. Lung Function

FEV1.0 was measured by spirometry at the Department of Clinical Physiology, Skane University Hospital in Lund, following the guidelines from the American Thoracic Society [25]. The results were expressed as proportion of predicted values (FEV1.0% pred.) calculated according to Quanjer et al. [26] from the patients' height, age, and sex. In case the patient did not perform any follow-up spirometry at the Department of Clinical Physiology (*n* = 6), the lung function was measured during a normal, clinical visit, and the result closest in time to the 10-year follow-up date was registered.

### 2.3. Bacterial Colonization

Samples for respiratory secretion cultures were taken when the patient attended a routine outpatient visit. Bacterial colonization with* P. aeruginosa* was defined at enrolment according to the Leeds criteria, using historical microbiology results from patient records and from the database at the Department of Microbiology. Patients were grouped in Leeds class 1 (chronic), Leeds class 2 (intermittent), Leeds class 3 (free of earlier colonization), and Leeds class 4 (never colonized with* P. aeruginosa*) [27]. Other bacteria detected included* Staphylococcus aureus, Hemophilus influenzae*,* Stenotrophomonas maltophilia,* and other Gram-negative bacteria such as* Escherichia coli.* There were no patients with methicillin resistant* Staphylococcus aureus* (MRSA). One patient, classified as Leeds class 3, was chronically colonized with* Burkholderia multivorans*. In Leeds group 1, twenty-one patients had no other bacteria than* P. aeruginosa*, twenty had* P. aeruginosa* and* Staphylococcus aureus* (in some cases with a third additional isolate), and three had* P. aeruginosa* and* Stenotrophomonas maltophilia*. The pattern of bacteria found in the different Leeds groups followed the expected path in CF patients: early colonization with* Hemophilus influenzae*, followed by* Staphylococcus aureus*, later* P. aeruginosa*, on its own or cohabituating with* Staphylococcus aureus, Hemophilus influenzae,* and in some cases* Stenotrophomonas maltophilia*.

### 2.4. Detection of Antibodies against BPI

IgA BPI-ANCA was analysed with ELISA and measured at the time of inclusion. Purified BPI was obtained from Wieslab AB (Lund, Sweden) and direct binding was performed [28]. In short, antigens were coated onto microtiter plates at a concentration of 1 microliter/mL. Serum samples were diluted 1/80 and incubated for one hour. Bound antibodies were detected using alkaline phosphatase-conjugated goat anti-human IgA. BPI-ANCA was quantified from a calibrator curve that was serially diluted and the results were expressed as arbitrary units (U).

### 2.5. *Pseudomonas aeruginosa* Serology

*P. aeruginosa* serologies were analysed using anti-*Pseudomonas* IgG EIA, E15 from Mediagnost, Reutlingen, Germany, and antibodies against three exoproteins; alkaline protease (AP), Exotoxin A (ExoA), and Elastase (ELA) were measured at the time of inclusion. The test is a sandwich enzyme immunoassay. Serum or plasma samples are diluted and added to the wells of microtiter plates, which have been previously coated with the* PsA* antigens AP, ELA, or ExoA. Specific antibodies in the sample bind to the antigens present during an incubation of 2 hours at 37°C. After washing, the conjugate (anti-human IgG peroxidase labelled immunoglobulin) is added and incubated again for 2 hours at 37°C. After a final washing step, substrate is added and further incubated for 30 minutes at room temperature. The reaction is terminated on addition of stop solution accompanied by a change from blue to yellow. The absorbance of the colour reaction product is measured on a microtitre plate reader. Kappler et al. have investigated specificity and sensitivity in 2006 [10].

### 2.6. Statistical Methods

Pearson correlation coefficient (*r*) was used to examine and compare serology and BPI-ANCA in relation to bacterial colonization, lung function, future colonization, and long time outcome. Receiver operator curves (ROC) were generated to graphically illustrate sensitivity and specificity for these assays. Area under curve (AUC) with 95% confidence interval (CI) was also calculated.

## 3. Results

### 3.1. Baseline Characteristics

The baseline characteristics of the 117 patients included in the study are presented in Table 1. The median age at inclusion was 19 years (IQR 11–25 years). 65 patients were homozygous for ΔF508, 46 were heterozygous (in 4 of these patients the other mutation was unknown), and 6 had other mutations. The majority of the patients were pancreatic insufficient (101 patients, 86%), 13 were pancreatic sufficient, and additionally 3 patients were born pancreatic sufficient but had developed pancreatic insufficiency (mainly due to recurrent pancreatitis).

Out of the 117 patients 48 (31%) were classified as chronically colonized with* PsA* (Leeds class 1). The median age in this group was 21 years (IQR 17–27) and 14 patients were below the age of 18.

### 3.2. Lung Function Impairment Correlated Better with BPI-ANCA than with the Anti-*Pseudomonas* Serologies

**Figure 1:**
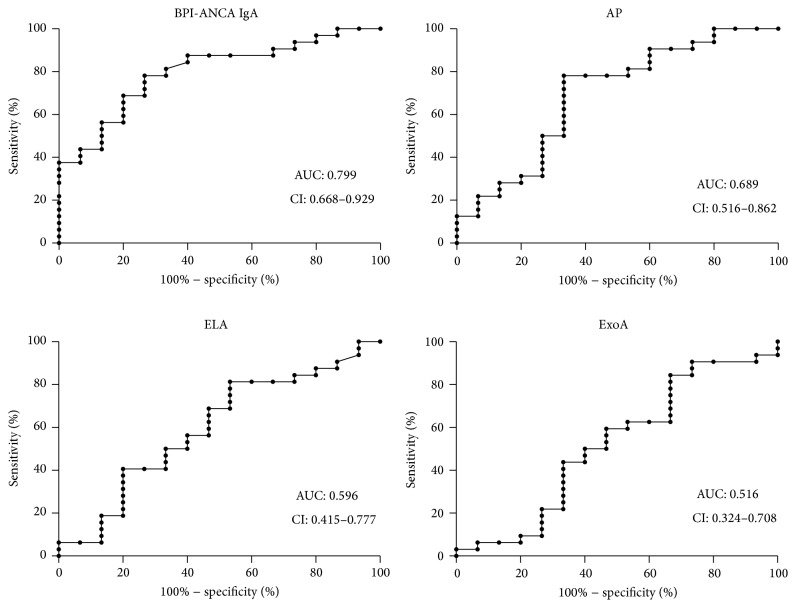
*P. aeruginosa* related lung damage. ROC-curves comparing how well the different tests identify patients chronically colonized with* P. aeruginosa* with an FEV1.0 below 80% of predicted (*n* = 32). IgA BPI-ANCA was the best test for this purpose, with an area under curve of 0.8 (CI: 0.688–0.929, *p* value 0.001).

The median lung function in the whole cohort at inclusion was 84% of predicted FEV1 (IQR 60–96%). The lung function varied in the different colonization groups where 47 patients in Leeds 1 (chronic) had a median lung function of 62% of predicted FEV1 at inclusion (IQR 41–85) (one patient was too young to perform spirometry). The lung functions in the Leeds groups 2, 3, and 4 (intermittent, free, and never colonized) were 95%, 90%, and 89% (FEV1.0% pred.), respectively (Table 1). The correlation between lung function impairment (100-FEV1.0%) and IgA BPI-ANCA in the chronically colonised group gave an *r*-value of 0.44. A value in the same range was achieved with the anti-AP test (*r* = 0.35), while a lesser degree of correlation was seen for the anti-ELA test (*r* = 0.20) and hardly any with the anti-ExoA test (*r* = 0.06) (Table 3). Another way of expressing the same finding is depicted in Figure 1, which presents ROC-curves for the ability to detect lung function impairment (FEV1.0 < 80% pred.); IgA BPI-ANCA exhibited the highest value (AUC 0.799) while the corresponding values for the three bacterial serology tests ranged from 0.516 to 0.689.

### 3.3. Long Term Outcome

In the whole cohort, 25 patients (18%) died or were lung transplanted during the 10-year follow-up. In the chronically colonized group (Leeds 1) 20 patients (33%) either died or were lung transplanted. In the Leeds groups 2, 3, and 4 only five patients (1, 1, and 3, resp.) died or were lung transplanted. One of the patients in Leeds group 4 died from a non-CF related accident. At follow-up the remaining 28 patients in the chronically colonized group had a lung function of 61% of predicted FEV1 (IQR 50–76). In Leeds groups 2, 3, and 4 the follow-up lung functions were 86%, 69%, and 88%, respectively (Table 2).

The 20 patients who died or were transplanted in Leeds group 1 had a mean BPI-ANCA IgA level of 500 U at inclusion compared with 108 U (*p* = 0.025) for the 28 surviving without lung transplantation. The corresponding values for anti-PsA serology tests were as follows: AP 2612 versus 1713 (*p* = 0.038); ELA 4216 versus 2416 (*p* = 0.06); and ExoA 2598 versus 2416 (*p* = 0.41) and ROC analysis revealed AUC of 0.77 for BPI-ANCA (*p* = 0.002), 0.7 for AP (*p* = 0.02), 0.65 for ELA (*p* = 0.09), and 0.54 for ExoA (*p* = 0.6).

### 3.4. Identifying Patients with Chronic PsA Colonization

**Figure 2:**
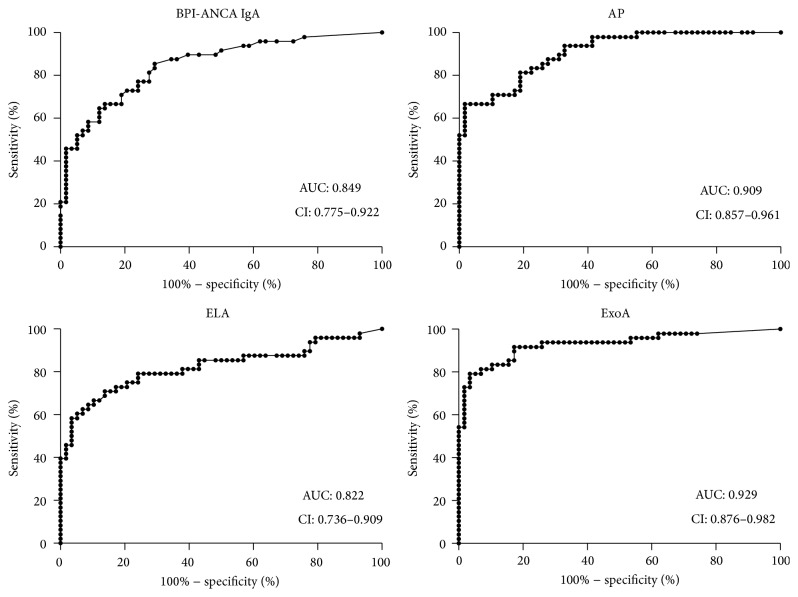
Identifying chronically colonized patients. ROC-curves of IgA BPI-ANCA and three different anti-*P. aeruginosa* serologies comparing their ability to identify patients with chronic* P. aeruginosa* colonization (*n* = 48). Controls were patients belonging to Leeds class 3 (free from earlier* P. aeruginosa*) or 4 (never had* P. aeruginosa*) (*n* = 58). Leeds 2 patients (intermittently colonized, *n* = 11) were omitted in this analysis. All tests were useful for this purpose, Exotoxin A presenting the best area under curve with 0.929 (CI: 0.876–0.982).

BPI-ANCA and the three different* P. aeruginosa* serology tests were all useful for identifying patients with chronic* P. aeruginosa* (AUC between 0.822 and 0.929), and there were no statistical differences between the tests. Among the chronically colonized (Leeds 1) patients the values obtained with the three* P. aeruginosa* serology tests correlated better with each other (*r*-values: 0.37; 0.46; and 0.58) than they did with the levels of IgA BPI-ANCA (*r*-values: 0.02, 0.12, and 0.21). The ability of the different serological tests to classify patients as being colonized is depicted in Figure 2.

### 3.5. Detection of Future Colonization Was Not Possible with Any of the Tests

**Figure 3:**
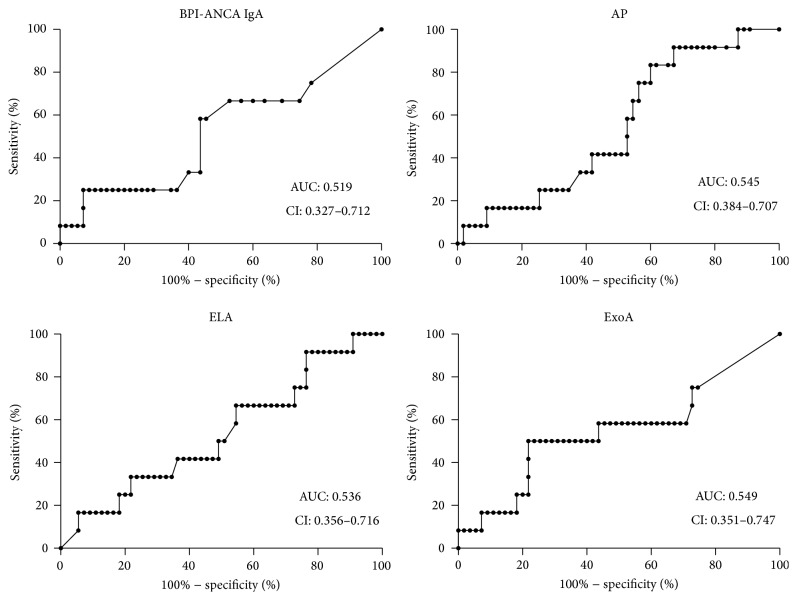
Prediction of future colonization with* P. aeruginosa*. Patients (*n* = 12) who changed colonization status to chronic (Leeds 1) within three years were identified. Controls were patients staying in Leeds class 2, 3, or 4 (intermittent, free, or never colonized with* P. aeruginosa*). ROC-curves were generated to evaluate if any of the four tests (IgA BPI-ANCA, AP, ELA, or ExoA) was able to find these patients three years before the chronic colonization was established. None of the tests could be used for that purpose.

To examine the ability of the different tests to detect subclinical colonization, heralding future permanent colonization, we compared values among those who during a follow-up of three years changed their colonisation status from Leeds 2, 3, and 4 to Leeds 1. Twelve patients underwent such a change. None of the tests were able to identify such patients (Figure 3).

## 4. Discussion

The major findings in the present study are that BPI-ANCA exhibits a better correlation with* P. aeruginosa* induced lung function impairment and future adverse events and has a similar capacity to detect chronic colonization as standard anti-*Pseudomonas *serology.

BPI-ANCA seems to develop in response to* P. aeruginosa *colonization, but there are also patients colonized with* P. aeruginosa *who do not develop BPI-ANCA [22, 24]. After eradication of* P. aeruginosa *colonization by lung transplantation a significant decrease in BPI-ANCA levels has been seen [22] and it has been shown that BPI-ANCA levels significantly decrease after sinus surgery [29].

The relationship between lung function and BPI-ANCA in the present cohort has previously been published [22]. Anti-*P. aeruginosa *serology is generally not used as a way to evaluate the CF patients' lung function, but Winnie and Cowan did find a strong inverse relationship between anti-*P. aeruginosa *titre and pulmonary function test in patients chronically colonized with* P. aeruginosa* [30]. West observed a relationship between enhanced antibody titers against* P. aeruginosa* and clinical status, up to 6 months before the first positive* P. aeruginosa* culture [8]. In the present study we show that at least the anti-AP test showed a significant correlation with decreased lung function. However, the *r*-value was higher with BPI-ANCA. In the present study 20 of the 48 chronically colonized patients experienced an adverse outcome (death or lung transplantation). BPI-ANCA had a higher capability of predicting these end-points (AUC = 0.77, *p* = 0.002) compared with serology tests: AUC for AP 0.7 P (*p* = 0.02), ELA 0.65 (*p* = 0.09), and ExoA 0.54 (*p* = 0.6). This finding is in line with earlier results from our group [24], where BPI-ANCA level, in contrast to Leeds class, was significantly correlated with outcome. These findings suggest that BPI-ANCA is a biomarker of an unfavourable host-pathogen interaction and not merely a substitute marker for* P. aeruginosa* colonization.

The present study indicates that BPI-ANCA has the same ability as anti-*P. aeruginosa* serology to detect chronic colonisation. As different serology tests correlated better with each other than with BPI-ANCA, it might be meaningful to include BPI-ANCA in future serology panels to increase the diagnostic yield. This, however, requires more studies on larger cohorts to determine. In this study we have used the commercially available analysis kit, analysing ELA, ExoA, and AP. There are other tests available that exhibit similar or even better sensitivity and specificity, but this kit is well known among CF clinics and regularly used [11].

Out of the 69 patients, who, at inclusion, were not chronically colonized with* P. aeruginosa*, twelve changed their colonization status to chronic (Leeds 1) within three years. Out of these patients, three were categorized as intermittently colonized (Leeds 2), three were free of earlier* P. aeruginosa, *and six had never had* P. aeruginosa*. None of the tests compared in this study was able to predict which patients were to develop chronic* P. aeruginosa* colonization. It is possible that three years to change to chronic colonization was too long and that we would see other results if we had shortened this time frame as antibodies against* P. aeruginosa* have been shown to correlate with success of long term eradication [31] and risk of recurrent* P. aeruginosa* isolation after initial eradication [32].

So far, BPI-ANCA is not commonly used in CF clinics. In this study we have investigated what distinguishes BPI-ANCA from the much more used anti-*P. aeruginosa* serology. We show that BPI-ANCA is a better tool for identifying patients, chronically colonized with* P. aeruginosa*, who have developed lung function impairment, indicating that BPI-ANCA means something more than merely the presence of* P. aeruginosa* ROC generated showed a good specificity and sensitivity for both BPI-ANCA and the different serologies when it came to identifying patients with a chronic* P. aeruginosa* colonisation. Neither BPI-ANCA nor any of the studied serology tests were able to inform us if a patient was going to develop a chronic* P. aeruginosa* colonisation within the next three years.

The main objective of this study was to compare if BPI-ANCA and anti-*Pseudomonas* serologies show the same things. We conclude that all investigated tests are able to identify patients with a chronic* PsA* colonization/infection to a similar extent, but as hypothesised, BPI-ANCA indicates that a more profound negative development has occurred in the* P. aeruginosa* infected lung. The reason why this happens is still not known and further studies are needed to explore this negative host/pathogen interaction.

## Figures and Tables

**Table 1:** Description of the cohort at enrolment.

	Chronic PsA Leeds 1	Intermittent PsA Leeds 2	Free from PsA Leeds 3	Never had PsA Leeds 4	Total
*n *	48	11	16	42	117

Median age (IQR) *years *	21 (17–27)	17 (10–23)	15 (7–22)	16 (7–25)	19 (11–25)

Sex m/f *n*	27/21	4/7	5/10	24/18	60/57

Mutation ΔF508/ΔF508 ΔF508/other Other/other ΔF508/unknown *n*	28/17/2/1	7/4/0/0	10/5/1/0	20/16/3/3	65/42/6/4

Pancreatic insufficiency *n* (%)	47 (98%)	8 (73%)	14 (87,5%)	31 (74%)	100 (85%)

Median lung function (IQR) *FEV% pred. *	62% (41–85)	95% (90–101)	90% (74–102)	89% (80–104)	(*n* = 112) 84% (60–96)

**Table 2:** Results.

	Chronic PsA Leeds 1	Intermittent PsA Leeds 2	Free from PsA Leeds 3	Never had PsA Leeds 4	Total
Alkaline protease Median (IQR*) ELISA units *	2056 (596–3372)	327 (130–661)	325 (162–593)	194 (46–381)	450 (167–1299)

Elastase Median (IQR)* ELISA units *	1696 (439–5660)	421 (170–2205)	284 (197–383)	239 (152–383)	389 (217–1750)

Exotoxin A Median (IQR)* ELISA units *	1985 (890–3925)	451 (99–839)	224 (56–517)	117 (0–225)	358 (103–1410)

BPI-ANCA IgA Median (IQR) *ELISA units *	93 (28–2225)	8 (4–22)	7 (1–18)	8 (1–30)	21 (5–89)

Change to Leeds 1 within 3 years *n*	na	3	3	6	12

Lung transplantation or death within 10 years *n* (%)	20 (33%)	1 (9%)	1 (6%)	3 (7%)^1^	25 (18%)

Lung function at 10 years follow-up Median (IQR) *FEV1% pred. *	61,5% (50–76)	86% (76–101)	69% (47–83)	88% (71–101)	76% (60–94)

^1^One of these patients died due to a non-CF related cause.

**Table 3:** Correlations. Patients with a chronic colonization with PsA (Leeds 1) and known lung function at inclusion (*n* = 48).

Correlates with	BPI-ANCA IgA	Alkaline protease	Elastase	Exotoxin A
Lung function decline (100-FEV1% pred.) *r*-value	0.44	0.35	0.20	0.06

Alkaline protease *r*-value	0.12		0.37	0.58

Elastase *r*-value	0.21	0.37		0.46

Exotoxin A *r*-value	0.02	0.58	0.46	

BPI-ANCA IgA *r*-value		0.12	0.21	0.02
